# Engineered Accumulation of Bicarbonate in Plant Chloroplasts: Known Knowns and Known Unknowns

**DOI:** 10.3389/fpls.2021.727118

**Published:** 2021-08-31

**Authors:** Sarah Rottet, Britta Förster, Wei Yih Hee, Loraine M. Rourke, G. Dean Price, Benedict M. Long

**Affiliations:** ^1^Realizing Increased Photosynthetic Efficiency (RIPE), The Australian National University, Canberra, ACT, Australia; ^2^Australian Research Council Centre of Excellence for Translational Photosynthesis, Research School of Biology, The Australian National University, Canberra, ACT, Australia

**Keywords:** CO_2_-concentrating mechanism, bicarbonate transport, chloroplast envelope, improving photosynthesis, chloroplast engineering

## Abstract

Heterologous synthesis of a biophysical CO_2_-concentrating mechanism (CCM) in plant chloroplasts offers significant potential to improve the photosynthetic efficiency of C_3_ plants and could translate into substantial increases in crop yield. In organisms utilizing a biophysical CCM, this mechanism efficiently surrounds a high turnover rate Rubisco with elevated CO_2_ concentrations to maximize carboxylation rates. A critical feature of both native biophysical CCMs and one engineered into a C_3_ plant chloroplast is functional bicarbonate (HCO_3_^−^) transporters and vectorial CO_2_-to-HCO_3_^−^ converters. Engineering strategies aim to locate these transporters and conversion systems to the C_3_ chloroplast, enabling elevation of HCO_3_^−^ concentrations within the chloroplast stroma. Several CCM components have been identified in proteobacteria, cyanobacteria, and microalgae as likely candidates for this approach, yet their successful functional expression in C_3_ plant chloroplasts remains elusive. Here, we discuss the challenges in expressing and regulating functional HCO_3_^−^ transporter, and CO_2_-to-HCO_3_^−^ converter candidates in chloroplast membranes as an essential step in engineering a biophysical CCM within plant chloroplasts. We highlight the broad technical and physiological concerns which must be considered in proposed engineering strategies, and present our current status of both knowledge and knowledge-gaps which will affect successful engineering outcomes.

## Introduction

Crop improvement technologies utilizing synthetic biology approaches have been central to a number of recent advances in photosynthetic output (e.g., Kromdijk et al., 2016; Salesse-Smith et al., 2018; Ermakova et al., 2019; South et al., 2019; Batista-Silva et al., 2020; López-Calcagno et al., 2020). These ambitious aims come at an unprecedented time in human history when agricultural productivity must be rapidly boosted in order to feed future populations (Kromdijk and Long, 2016). In a 2008 review, we discussed the potential of utilizing components of the CO_2_-concentrating mechanism (CCM) of cyanobacteria as a means to improve crop photosynthetic CO_2_ fixation (Price et al., 2008), with potential to raise rates of carboxylation at ribulose-1,5-bisphosphate (RuBP) carboxylase/oxygenase (Rubisco) while improving nitrogen and water-use efficiencies (Price et al., 2011a; McGrath and Long, 2014; Rae et al., 2017). In the intervening years great steps forward have been made to address this challenge, yet many uncertainties remain on the path to generating a functional chloroplastic CCM.

**Figure 1:**
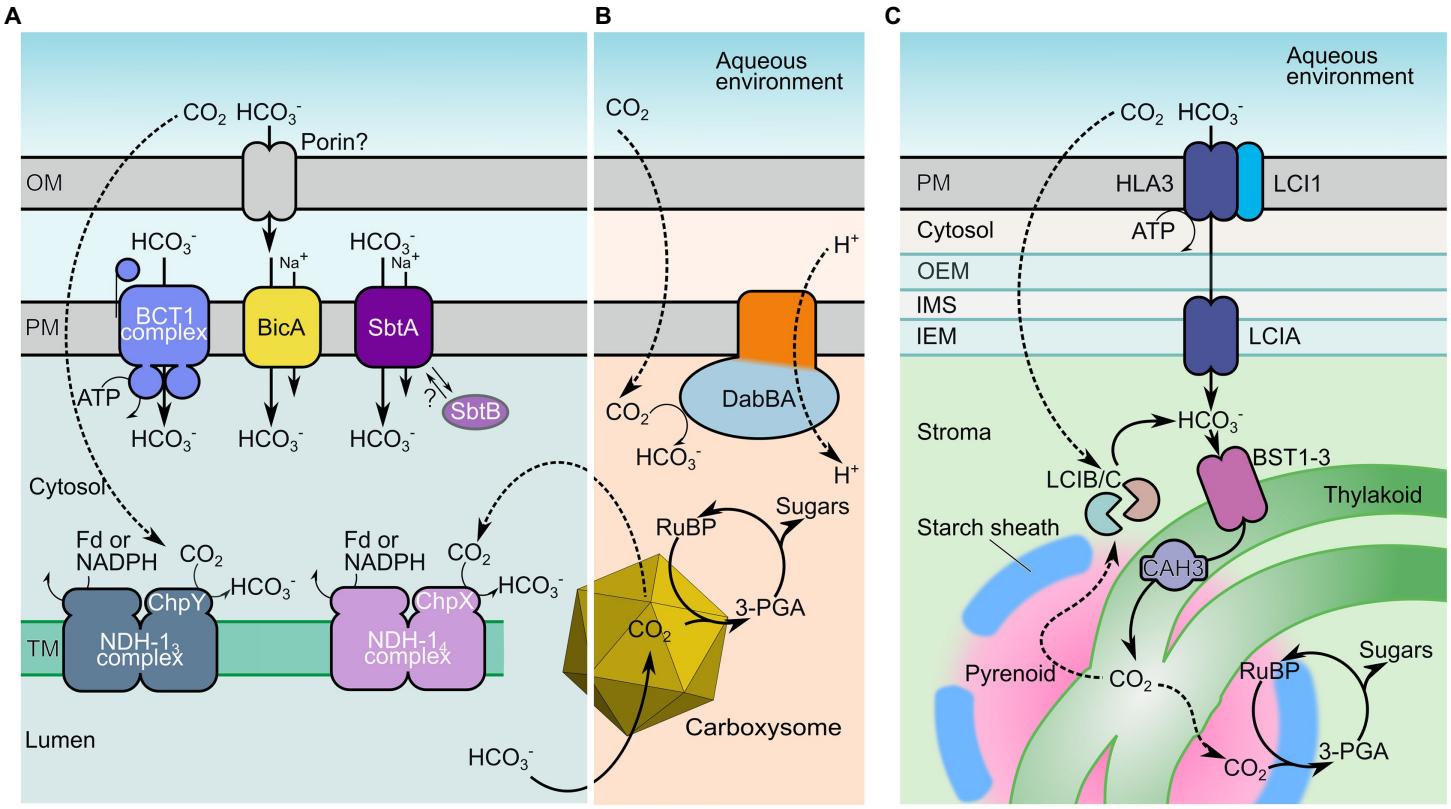
Inorganic carbon uptake components of cyanobacterial, proteobacterial, and microalgal CO_2_-concentrating mechanisms (CCMs). Key inorganic carbon transport mechanisms of cyanobacteria **(A)**, proteobacteria **(B)**, and microalgae **(C)** that facilitate elevated cytoplasmic and stromal HCO_3_^−^ concentrations. The HCO_3_^−^ pool is utilized to generate localized high concentrations of CO_2_ in specialized Rubisco-containing compartments knowns as carboxysomes **(A,B)** or pyrenoids **(C)**, supporting high carboxylation rates. In cyanobacteria **(A)**, HCO_3_^−^ is potentially supplied to the periplasmic space *via* an outer-membrane (OM) porin, and is directly transferred across the plasma membrane (PM) by the single-protein Na^+^-dependent transporters bicarbonate transporter A (BicA) and SbtA, or by the ATP-driven complex BCT1. In addition, cytosolic CO_2_, acquired by either diffusion, leakage from the carboxysome or spontaneous dehydration of HCO_3_^−^, is converted to HCO_3_^−^ by the energy-coupled, vectorial CO_2_ pumps NHD-1_3_ and NHD-1_4_ in the thylakoid membranes (TM). In proteobacteria **(B)**, DabBA plays a similar role, taking advantage of relatively high rates of CO_2_ influx from a low-pH external environment to vectorially generate a cytoplasmic HCO_3_^−^ pool (Desmarais et al., 2019). In microalgae (C), HCO_3_^−^ is accumulated *via* a series of transporters located on the PM (LCI1 and presumably ATP-driven HLA3), the chloroplast inner envelope membrane [inner-envelope membrane (IEM); LCIA] and the TM (bestrophins, BST1-3). Thylakoids traverse the Rubisco-containing pyrenoid where the thylakoid lumen-localized carbonic anhydrase (CA) CAH3 is thought to convert HCO_3_^−^ supplied to the thylakoid lumen to CO_2_. Analogous to the cyanobacterial system, the LCIB/C complex constitutes a putative, vectorial CA that may recycle any CO_2_ arising in the chloroplast stroma back to HCO_3_^−^. Fd, ferredoxin; RuBP, ribulose-1,5-bisphosphate; 3-PGA, 3-phosphoglycerate; SbtB, SbtA regulator protein; and ChpX/ChpY, NDH-1 complex vectorial CO_2_-HCO_3_^−^ domains. Individual transporter proteins are as listed in Table 1.

The CCMs of proteobacteria, cyanobacteria, and microalgae are comprised of bicarbonate (HCO_3_^−^) transporters and vectorial CO_2_-to-HCO_3_^−^ conversion complexes which, in concert, accumulate a high concentration of HCO_3_^−^ in prokaryotic cells and microalgal chloroplasts (Figure 1; Kaplan et al., 1980; Badger and Price, 2003; Moroney and Ynalvez, 2007). As a charged species of inorganic carbon (C_i_), HCO_3_^−^ is not freely diffusible through cell membranes (Tolleter et al., 2017), and allows for the generation of an elevated cellular or stromal HCO_3_^−^ pool compared with the external environment (Price and Badger, 1989a). The second chief component of these CCMs are specialized Rubisco compartments called carboxysomes (Rae et al., 2013) and pyrenoids (Figure 1; Moroney and Ynalvez, 2007; Mackinder, 2018; Hennacy and Jonikas, 2020) where co-localized carbonic anhydrase (CA) enzymes dehydrate HCO_3_^−^ into CO_2_, providing high concentrations of CO_2_ as substrate for RuBP carboxylation.

Collectively, these systems are often termed biophysical CCMs since their function utilizes the active movement of C_i_ across cellular compartments to release it as CO_2_ around Rubisco (Giordano et al., 2005). This is distinct from biochemical CCMs found in C_4_ and CAM plants, which generally utilize HCO_3_^−^ for the carboxylation of phosphoenolpyruvate into transportable organic acids, prior to spatial or temporal CO_2_ re-release and carboxylation by Rubisco.

Modeling has shown that the installation of biophysical CCM HCO_3_^−^ transporters in the inner-envelope membrane (IEM) of C_3_ chloroplasts is sufficient to elevate the chloroplastic C_i_, leading to a net improvement in CO_2_ available for Rubisco carboxylation and therefore net carbon gain (Price et al., 2011a; McGrath and Long, 2014). This initial step in the conversion of crop plant chloroplasts to a sub-cellular CCM not only provides potential yield gains but is also necessary to generate the required stromal [HCO_3_^−^] needed for carboxysome or pyrenoid function in the engineering trajectory toward a complete chloroplastic CCM (Price et al., 2013). Therefore, the successful engineering of HCO_3_^−^ accumulation in C_3_ stroma is a critical step in this process.

While the idea to generate a C_3_ chloroplastic CCM has been considered for some time (Price et al., 2008), the pace of progress in this field highlights a myriad of conceptual and technical challenges associated with achieving such a complex goal. Progress toward the construction of carboxysomes and pyrenoids in C_3_ chloroplasts has been made (Lin et al., 2014a; Long et al., 2018; Atkinson et al., 2020), and the transfer of a complete and functional CCM from proteobacteria into Rubisco-dependent *Escherichia coli* (Flamholz et al., 2020) indicates theoretical potential for successful transfer of CCMs to plants. However, hurdles remain in both understanding and constructing CCM components within eukaryotic organelles where system complexity confounds an already difficult engineering task. This is exemplified by reports of the successful expression of HCO_3_^−^ transporters into C_3_ chloroplasts, but their lack of function and/or incorrect targeting (Pengelly et al., 2014; Atkinson et al., 2016; Rolland et al., 2016; Uehara et al., 2016, 2020), or lack of functional characterization *in planta* (Nölke et al., 2019), highlights the need to further understand the composite interactions of chloroplast protein targeting, membrane energization, and small molecule passage across the chloroplast envelope.

Herein, we discuss some of the known complexities associated with the engineering task of generating functional HCO_3_^−^ transport systems in C_3_ chloroplasts and highlight unknown details, which require ongoing research focus to enable a clearer path to successful elevation of chloroplastic HCO_3_^−^ for increased carboxylation efficiency in crop plants.

## Can Hco_3_^−^ Concentrations Be Elevated In A C_3_ Chloroplast?

The terrestrial nature of C_3_ plants and their appearance in geological history during a period of relatively high atmospheric CO_2_ (Flamholz and Shih, 2020) is a possible contributor to the absence of biophysical CCMs from higher plant chloroplasts (Raven et al., 2017). The efficiency of Rubisco carboxylation is hampered by O_2_, leading to photorespiratory expenditure of accumulated CO_2_ and chemical energy (Busch, 2020). It is assumed that factors selecting for maintenance of relatively high rates of carboxylation, as atmospheric concentrations of CO_2_ decreased while O_2_ increased approximately 350 million years ago, may have led to a divergence in mechanistic adaptations between aquatic and terrestrial photosynthetic organisms (Flamholz and Shih, 2020; Long et al., 2021). Thus, cyanobacteria and many eukaryotic algae evolved CCMs to overcome these challenges, while emerging terrestrial C_3_ plants have maintained a larger investment in Rubisco and evolved to maximize beneficial biochemical contributions from photorespiratory nitrogen and sulfur metabolism (Shi and Bloom, 2021). As a result, terrestrial C_3_ plant lineages have not evolved with a capability to elevate chloroplastic C_i_ concentrations like many of their aquatic counterparts. Indeed, there is good argument that biochemical CCM evolution (e.g., C_4_ photosynthesis) would be favored in terrestrial systems over strategies which accumulate HCO_3_^−^ (Flamholz and Shih, 2020). While horizontal gene transfer may have been involved in the evolution of C_4_ photosynthesis (Wickell and Li, 2020), there has presumably been very little opportunity or evolutionary pressure for plants to acquire genes from aquatic biophysical CCMs in order to evolve alternative CO_2_ fixation strategies. In addition, the slower diffusion of C_i_ species in aquatic environments compared with plant tissue may confine evolutionary trajectories (Raven et al., 2017; Flamholz and Shih, 2020). This underscores the fact that the C_3_ chloroplast has evolved in a gaseous atmosphere and with alternative solutions to Rubisco promiscuity to its aquatic cousins, highlighting that the concept of an engineered chloroplastic CCM is one in which considerable evolutionary complexity must be considered.

When considering any engineering design for enhanced HCO_3_^−^ uptake into C_3_ chloroplasts, a reasonable question to ask is whether HCO_3_^−^ can be elevated in this organelle, and if so, how? There is sufficient HCO_3_^−^ in the mesophyll cytoplasm available for transport into chloroplasts (at least 250μM; Evans and von Caemmerer, 1996). However, a CCM engineering strategy must ensure HCO_3_^−^ can gain passage across both the outer-envelope membrane (OEM) and IEM of the chloroplast. Given that C_3_ chloroplasts typically access C_i_ from the external environment (primarily as the more membrane-permeable CO_2_); chloroplast membranes appear not to have specific HCO_3_^−^ transport mechanisms (Rolland et al., 2012). Nonetheless, a number of oxyanions, such as phosphate, nitrate, and sulfate evidently do diffuse through the OEM (Bölter et al., 1999). Notably, simple diffusion of CO_2_ through leaf tissue is insufficient to support the supply rates needed for observed rates of CO_2_ assimilation by plants (Morison et al., 2005), and it is likely that CO_2_ entry into the chloroplast is also facilitated by CO_2_-permeable aquaporins (Flexas et al., 2006; Evans et al., 2009; Tolleter et al., 2017; Ermakova et al., 2021) and CA-driven distribution of C_i_ between predominant species (HCO_3_^−^ and CO_2_; Price et al., 1994). Therefore, the facilitated entry of C_i_ into C_3_ chloroplasts is conceptually not counter to contemporary chloroplast function, and on face value would appear beneficial.

**Figure 2:**
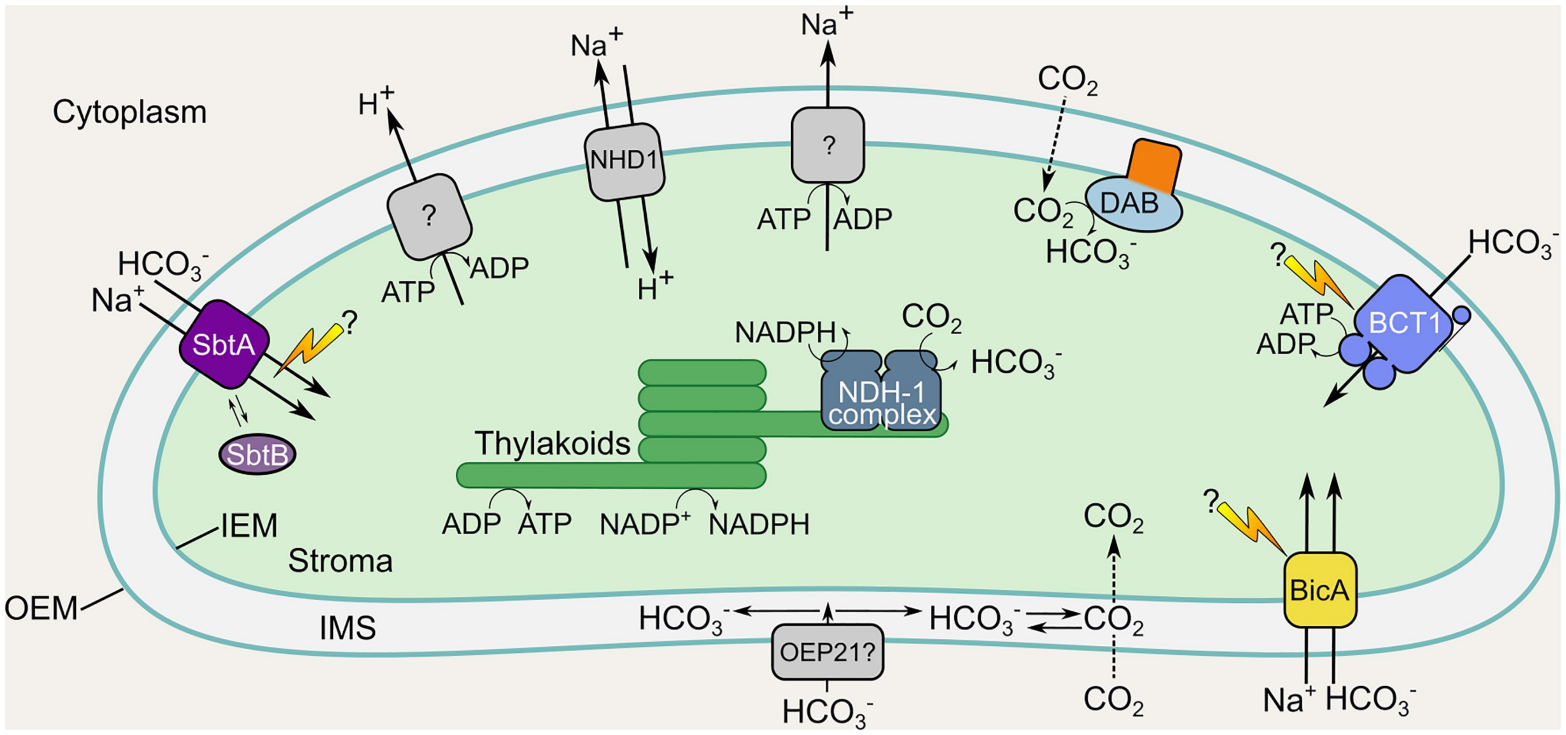
Location and function of inorganic carbon uptake systems in a proposed chloroplastic CCM. Inorganic carbon uptake components from cyanobacteria and proteobacteria proposed for engineering into the chloroplast IEM and thylakoids of terrestrial C_3_ plants. Membrane transporters and vectorial CO_2_-HCO_3_^−^ conversion complexes from cyanobacteria and proteobacteria are shown in color (see Figure 1; Table 1). Additional native chloroplastic systems which may support the required energetics and facilitation of active HCO_3_^−^ uptake (including Na^+^ and H^+^ extrusion systems and the NHD1 Na^+^/H^+^ antiporter) are shown in gray. The cyanobacterial HCO_3_^−^ transporters BicA and SbtA, as well as the ATP-driven BCT1 complex are targeted to the IEM may require unknown activation processes, indicated by lightning bolts. Note the added complexity of the multi-subunit BCT1 complex requiring protein components in the inter-membrane space (IMS), IEM, and stroma. The cyanobacterial vectorial NDH-1 complex has been suggested on the thylakoid membrane as it likely requires energization by components of the photosynthetic electron transport chain (ETC). The proteobacterial vectorial DAB complex is tentatively depicted on the IEM, but, alternatively, co-localization with the ETC on the thylakoid membrane may be favorable for its energization. The broad-specificity channel protein OEP21 on the outer-envelope membrane (OEM) is depicted as a putative access point for cytoplasmic HCO_3_^−^ uptake into the IMS. Individual transporter proteins are as listed in Table 1.

In general, solute transport across the chloroplast OEM is considered to be relatively unhindered due to the presence of low-selectivity and large-molecule channel proteins present in this membrane (Bölter et al., 1999; Hemmler et al., 2006; Duy et al., 2007). It is expected that anion passage into the inter-membrane space (IMS), and presumably that of HCO_3_^−^, occurs *via* at least one of the outer envelope protein channels (OEPs; Duy et al., 2007), with OEP21 a potential route for broad anion uptake into the IMS (Figure 2; Hemmler et al., 2006). While inward passage through this specific channel may be hampered by triose-phosphate export in the light (Duy et al., 2007), it and other OEPs offer broad selective import into the IMS. Currently, there is no reason to expect that HCO_3_^−^ cannot access the IMS. Nonetheless, it is worthy of consideration, and additional transport mechanisms or solutions should be considered for the elevation of IMS [HCO_3_^−^] if this becomes a roadblock to the overall strategy. Notably, insertion of an IMS-specific CA would likely generate the requisite HCO_3_^−^ from diffusion of CO_2_ in this location (depending on the IMS pH) for utilization by an IEM-localized pump, in the unlikely scenario that insufficient HCO_3_^−^ is present here. The ΔpH across the chloroplast IEM has been measured to be up to 1 pH unit (Demmig and Gimmler, 1983) suggesting that an IMS pH of 7–7.5 is feasible in the light, ensuring that >80% of C_i_ species would exist as HCO_3_^−^ in the presence of CA.

Assuming sufficient HCO_3_^−^ is available in the IMS from the cytosolic pool, its transport across the IEM into the chloroplast stroma is predicted to be feasible using either high affinity, low flux transporters [e.g., the cyanobacterial sodium-dependent bicarbonate transporter, SbtA, and the ATP driven bicarbonate transporter, BCT1; Table 1; Figures 1, 2], or low to medium affinity, high flux transporters (e.g., BicA; Table 1; Figures 1, 2). For the most part, the affinity of these HCO_3_^−^ transporter types falls below the proposed cytosolic [HCO_3_^−^] (Table 1), suggesting that sufficient transport is feasible. Either independently, or in concert, modeling suggests that functional forms of these transporter types should provide net import into the stroma and enable increased CO_2_ supply to Rubisco (Price et al., 2011a).

**Table 1:** Inorganic carbon (C_i_) uptake systems relevant to expression of CCMs in chloroplasts.

C_i_ uptake system	Organism subcellular location	Functional units	Classification	Substrates; Energization	Kinetic properties	References
BicA	*Cyanobacteria*^a^ plasma membrane	Homodimer	Sulfate permease (SULP), Solute carrier family (SLC26A)	HCO_3_^−^/Na^+^ symport; dependent on Na^+^ gradient	Medium-high flux; low affinity (*k*_0.5_ 74–353μM HCO_3_^−^)	Price et al., 2004; Shelden et al., 2010; Price and Howitt, 2011; Wang et al., 2019
SbtA	*Cyanobacteria*^a^ plasma membrane	Possible homotrimer		HCO_3_^−^/Na^+^ symport; dependent on Na^+^ gradient	Low flux; high affinity (*k*_0.5_ 2–38μM HCO_3_^−^)	Price et al., 2004, 2011a,b; Du et al., 2014; Förster et al., 2021
BCT1 (*cmpABCD* operon)	*Cyanobacteria*^a^ plasma membrane	Five subunit complex: CmpA (substrate binding), 2x CmpB (TMD), CmpC (ATPase: substrate binding fusion), and CmpD (ATPase)	ATP-binding cassette (ABC) transporter	HCO_3_^−^; ATP hydrolysis^c^	Low flux; high affinity (*k*_0.5_ 10–15μM HCO_3_^−^)	Omata et al., 1999; Koropatkin et al., 2007; Price et al., 2011a
LCIA/Nar1.2	*Chlamydomonas*^b^ chloroplast envelope	Unknown	Formate-nitrite transporter family	HCO_3_^−^; unknown	Unknown	Wang et al., 2015; Atkinson et al., 2016
HLA3	*Chlamydomonas*^b^ plasma membrane	Unknown	ABC transporter	HCO_3_^−^; ATP hydrolysis^c^	Unknown	Gao et al., 2015; Wang et al., 2015; Atkinson et al., 2016
LCI1	*Chlamydomonas*^b^ plasma membrane	Unknown	Anion channel	Ci (some evidence for CO_2_); unknown	Unknown	Wang et al., 2015; Atkinson et al., 2016; Kono and Spalding, 2020
BST-1 BST-2 BST-3	*Chlamydomonas*^b^ thylakoid membrane	BST-1 pentamer	Bestrophin-like proteins, Anion/Cl^−^channel family	HCO_3_^−^; unknown	Unknown	Mukherjee et al., 2019
NHD-1_3_	*Cyanobacteria*^a^ thylakoid membrane	21 subunit complex: CupS, ChpY (CupA, type II β-CA), NdhD3, and F3 (specialized for CO_2_ hydration) NdhA, B, C, E, G-Q, S, V (NDH-1 core, antiporter-like H^+^ pumping proteins, Fd binding, PQ binding)	Specialized respiratory NDH-1-type complex, energy-coupled vectorial CA	CO_2_; photosynthetic electron transport/redox-coupled H^+^ pumping^c^, reduced Fd-dependent	Low flux; high affinity (*k*_0.5_ 1–2μM CO_2_)	Maeda et al., 2002; Price et al., 2011a; Laughlin et al., 2020; Schuller et al., 2020
NHD-1_4;_ *ndhA,B,C,D3,E,F3,G-Q,S,V* *chpX/cupB*	*Cyanobacteria*^a^ thylakoid membrane	20 subunit complex: ChpX (CupB, type II β-CA), NdhD4, and F4 (specialized for CO_2_ hydration) NdhA, B, C, E, G-Q, S, V (NDH-1 core, antiporter-like H^+^ pumping proteins, Fd binding, PQ binding)	Specialized respiratory NDH-1-type complex, Energy-coupled vectorial CA	CO_2_; photosynthetic electron transport/redox-coupled H^+^ pumping ^c^, reduced Fd-dependent	High flux; medium affinity (*k*_0.5_ 10–15μM CO_2_)	Maeda et al., 2002; Price et al., 2011a; Laughlin et al., 2020; Schuller et al., 2020
DAB2; *dabA2*, *dabB2*	*Halothiobacillus neapolitanus* plasma membrane	heterodimer: DabA2 (type II β-CA homolog), DabB2 (H^+^ pumping protein homolog)	Energy-coupled vectorial CA	CO_2_; cation gradient-coupled^c^	Unknown	Desmarais et al., 2019
LCIB/C; *lciB*, *lciC*	*Chlamydomonas*^b^ Chloroplast stroma, pyrenoid periphery	heterodimer: LciB-LciC (β-CA subtype)	Vectorial? CA	CO_2_; unknown	Unknown	Duanmu et al., 2009; Wang et al., 2015; Jin et al., 2016

CA, Carbonic anhydrase; Fd, ferredoxin; PQ, plastoquinone; k_0.5_, substrate concentration supporting half-maximum C_i_ transport activity; TMD, transmembrane domain.

a Identified and characterized in several species incl. *Synechococcus elongatus* PCC7942, *Synechocystis* sp. PCC6803, *Synechococcus* sp. PCC7002, and *Thermosynechococcus elongatus*.

b Identified in *Chlamydomonas reinhardtii*.

c Energization is to some extent speculative based on structural homology.

Once HCO_3_^−^ concentrations in the chloroplast are elevated, it is acknowledged that stromal CA is likely to prevent the desired function of a complete chloroplastic CCM, since its action in converting HCO_3_^−^ to CO_2_ transforms the C_i_ pool from one with low membrane permeability to one which can rapidly diffuse away from the site of fixation (Price et al., 2013; McGrath and Long, 2014). This would rob an engineered carboxysome (housing a Rubisco with relatively high *K_M_CO_2_*) of its primary C_i_ substrate, and ectopic CA is known to lead to a high-CO_2_-requiring phenotype in cyanobacteria (Price and Badger, 1989a). However, in the development of a simpler CCM with only functional HCO_3_^−^ uptake, stromal CA would provide the rapid, pH-driven development of CO_2_ needed in the chloroplast to supply additional CO_2_ to Rubisco. The net effect of such a system is the modest elevation of chloroplastic C_i_, which leads to enhanced CO_2_ availability at Rubisco (Price et al., 2011a; McGrath and Long, 2014).

It is relevant to consider what effects elevated stromal HCO_3_^−^ might have on chloroplast function beyond the capability of supplying increased CO_2_ to Rubisco. A role for HCO_3_^−^ as a proton acceptor during water oxidation has been proposed in photosystem II (PSII) function, with HCO_3_^−^ providing stabilizing and protective effects (Shevela et al., 2012). CO_2_ formation from HCO_3_^−^ at PSII occurs at a rate that correlates with O_2_ evolution at the donor side (somewhat slower at the acceptor side; Shevela et al., 2020). A simplistic viewpoint therefore is that greater quantities of stromal HCO_3_^−^ may support PSII function rather than having any negative effects, as appears to be the case for cyanobacteria and microalgae. This PSII property highlights potential conversion of HCO_3_^−^ to CO_2_ in an engineered chloroplastic CCM, however, and longer-term goals would be to generate systems which recycle stromal CO_2_ back to HCO_3_^−^, whether it is generated through PSII action, anaplerotic reactions, or indeed leakage from an engineered carboxysome or pyrenoid (Price et al., 2013). Nonetheless, CO_2_ losses *via* these processes are likely to be minimal within an engineering scheme utilizing a HCO_3_^−^ transporter and a carboxysome, benefiting only marginally from the addition of vectorial CO_2_-to-HCO_3_^−^ conversion complexes (McGrath and Long, 2014).

## Which C_i_ Uptake Systems Could Facilitate Chloroplastic Hco_3_^−^ Accumulation?

Inorganic carbon acquisition is an essential step in driving a biophysical CCM and for maximizing its efficiency. Acquisition of the predominant C_i_ species (CO_2_ and HCO_3_^−^) contributes to the accumulation of an intracellular/chloroplastic HCO_3_^−^ pool well above external C_i_ levels, with up to 1,000-fold increases observed in cyanobacteria (Price, 2011). This can only be achieved by active C_i_ uptake against a concentration gradient, requiring energy, as opposed to passive diffusional uptake through protein channels such as CO_2_ aquaporins (Uehlein et al., 2012; Li et al., 2015). Active C_i_ uptake systems can be divided into two categories, energy-coupled CAs (also known as vectorial CO_2_ pumps or CO_2_-to-HCO_3_^−^ conversion systems) and active HCO_3_^−^ transporters. A number of C_i_ transport systems have been identified through genetic screens of high CO_2_ requiring mutants in the microalga *Chlamydomonas* (Spalding, 2008; Fang et al., 2012), several cyanobacteria (Price and Badger, 1989b; Badger and Price, 2003; Price et al., 2008) and, recently non-photosynthetic, CO_2_-fixing γ-proteobacteria (Scott et al., 2018; Desmarais et al., 2019), summarized in Table 1 and Sui et al. (2020).

### Cyanobacterial C_i_ Uptake Systems

In cyanobacteria, five C_i_ uptake systems have been verified, subsets of which are present in all species (Figure 1A; Table 1). These transport systems differ in subcellular localization, substrate affinity, flux rates, energization and regulation of gene expression, and transport activity (Price, 2011). These properties somewhat determine their suitability for function in a proposed chloroplastic CCM. Dependent on the species, some C_i_ uptake systems are constitutively expressed, but in most cases, their expression is controlled by a combination of limiting C_i_ and light (Badger and Andrews, 1982; Kaplan et al., 1987; McGinn et al., 2003; Price et al., 2011b).

Intracellular CO_2_-to-HCO_3_^−^ conversion in cyanobacteria is facilitated by two specialized, thylakoid-located NAD(P)H dehydrogenase (NDH1) complexes related to the respiratory complex-I from mitochondria: the low C_i_-inducible, high affinity NDH-1_3_, and the constitutive, slightly lower affinity NDH-1_4_ complexes (Maeda et al., 2002; Ohkawa et al., 2002). The CO_2_ hydration subunits ChpY (CupA) and ChpX (CupB) of NDH-1_3_ and NDH-1_4,_ respectively, convert cytoplasmic CO_2_ to HCO_3_^−^, energized by reduced ferredoxin or NADPH that are generated by photosynthetic electron transport, and hence light-dependent (Ogawa et al., 1985; Maeda et al., 2002; Price et al., 2008; Battchikova et al., 2011). Recently, catalytic properties of the cryo-EM structure of the NDH-1_3_ complex have been analyzed applying quantum chemical density modeling to the cryo-EM structure, which has shed light onto putative regulatory mechanisms. CO_2_ hydration by NDH-1_3_ (and by analogy NDH-1_4_) is energetically linked to plastoquinone oxido-reduction coupled to proton-pumping, which controls the opening and closing of the putative CO_2_ diffusion channel and lateral removal of H^+^ generated in the CO_2_ hydration reaction catalyzed by the ChpY (CupA) subunit. This mechanism ensures that the backward reaction, and unfavorable CO_2_ release, is prevented (Badger and Price, 2003; Schuller et al., 2020). In plant chloroplasts, we expect such systems would require thylakoid localization for correct function.

Direct transfer of HCO_3_^−^ from the outside into the cytoplasm is facilitated by three types of plasma membrane-located HCO_3_^−^ transporters (Figure 1). The high affinity transporters, BCT1 and SbtA, were shown to be newly synthesized upon activation of HCO_3_^−^ uptake, while constitutively expressed BicA was induced without further *de novo* protein synthesis (Sültemeyer et al., 1998; McGinn et al., 2003). The heteromeric BCT1 complex (encoded by the *cmpABCD* operon; Table 1) is a high affinity-low flux HCO_3_^−^ transporter (Omata et al., 1999) of the ATP binding cassette (ABC) transporter superfamily, strongly suggesting ATP is used for energization. However, ATPase activity has not yet been demonstrated. BCT1 is composed of the membrane-anchored, substrate-binding protein CmpA, the homodimeric, membrane integral CmpB domain, and the cytoplasmic ATPase subunits CmpC and CmpD. CmpC appears to be a fusion protein which contains both the ATPase moiety and a putative regulatory substrate-binding domain homologous to CmpA. CmpA requires Ca^2+^ as co-ligand for binding of HCO_3_^−^, yet it is unclear whether Ca^2+^ plays a role in HCO_3_^−^ transport (Koropatkin et al., 2007). The complexity of the proposed subunit localization of BCT1 for chloroplast envelope expression (one subunit in the IMS, one in the IEM, and two in the stroma; see below) provides further plant engineering challenges in addition to correct transporter function.

Both, BicA and SbtA (Table 1) are HCO_3_^−^/Na^+^ symporters that require a cell-inward directed Na^+^ gradient for HCO_3_^−^ uptake (Shibata et al., 2002; Price et al., 2004), and as single protein transporters are attractive considerations for chloroplast engineering. BicA, a medium affinity-high flux transporter of the SLC26A solute carrier superfamily, is thought to function as a homodimer (Compton et al., 2011; Price and Howitt, 2014; Wang et al., 2019). The high affinity-low flux SbtA transporter, constitutes its own Na^+^-dependent HCO_3_^−^ transporter superfamily, and is likely to be active as a trimer (Du et al., 2014; Fang et al., 2021; Förster et al., 2021). These requirements for Na^+^ for HCO_3_^−^ uptake highlight the potential for excessive influx of Na^+^ in a chloroplast-based CCM which we discuss below.

### Non-photosynthetic Bacterial C_i_ Uptake Systems

The DAB proteins (encoded by the *dab1* and *dab2* operons) first identified in *Halothiobacillus neapolitanus* are distributed throughout prokaryotic phyla and have been proposed to function as energy-coupled CAs accumulating HCO_3_^−^ in the cytoplasm (Desmarais et al., 2019). A heterodimeric functional unit consists of the cytoplasmic exposed β-CA-like DabA protein coupled to the membrane-integral cation antiporter-like membrane subunit DabB (Figure 1B). Vectorial CO_2_ hydration by DabA has been hypothesized to be driven by a cation (H^+^ or Na^+^) gradient but has not yet been proven experimentally (Laughlin et al., 2020). From an engineering standpoint, DAB proteins may represent a viable alternative to NDH1_3/4_ complexes as candidates for CO_2_ uptake/recapture in chloroplasts as introduction of only two proteins is required for DABs compared to 20–21 different proteins for NDH1_3/4_ (Price et al., 2019). However, the suitability of DABs to function in chloroplasts will be uncertain until mechanisms of energization/regulation are resolved. In addition, we need to consider that DABs or any vectorial CA will only be effective in the final engineering stages once the endogenous stromal CA has been successfully removed (Price et al., 2011a).

### Microalgal C_i_ Uptake Systems

In *Chlamydomonas*, HCO_3_^−^ transporter genes induced under low C_i_ include plasma membrane-located HLA3 and LCI1 (Figure 2; Kono and Spalding, 2020), the chloroplast envelope-located LCIA (Nar1.2; Wang et al., 2011; Atkinson et al., 2016; Kono and Spalding, 2020), thylakoid membrane-integral bestrophin-like proteins BST1, BST2, and BST3 (Mukherjee et al., 2019), and the chloroplast-located CIA8 (Machingura et al., 2017). In addition, stromal LCIB/C complex and the thylakoid lumenal carbonic anhydrase CAH3 have been implied in CO_2_ recapture (reviewed in Mackinder, 2018; Mallikarjuna et al., 2020). Importantly, neither substrate affinities, net accumulation capacity, and energization nor regulatory mechanisms of individual transporters are sufficiently understood to evaluate their suitability for expression in C_3_ chloroplasts at this time (Table 1). It is highly likely though that HLA3 (Figure 1C), as a member of the ABC and transporter family, is energized by ATP hydrolysis (Wang et al., 2015), and heterologous expression of HLA3 or LCIA in *Xenopus* oocytes showed some HCO_3_^−^ uptake activity but were not characterized further (Atkinson et al., 2016).

## What are the Energetic and Functional Requirements of C_i_ Uptake Systems?

One major challenge for heterologous expression of C_i_ uptake systems is the regulation of protein function, which encompasses both primary energization and fine-tuning of activity to match dynamic photosynthetic CO_2_ assimilation capacity of plant leaves (Price et al., 2013; Rae et al., 2017; Mackinder, 2018). Irrespective of the organism, C_i_ uptake appears to be controlled at the level of gene expression as well as protein function. While our current knowledge allows us to control expression of transgenes quite effectively, control of protein function in a non-native environment is still vastly empirical and, without greater understanding, far from attaining control by rational design.

Regulation of transporter function appears to be as little understood as energization. Most knowledge has been gathered for the cyanobacterial C_i_ uptake systems (Table 1). In cyanobacteria, as in chloroplasts, elevated HCO_3_^−^ concentration is only beneficial for photosynthetic carbon gain in the light. For maximum efficiency, C_i_ uptake activity needs to be in tune with day/night cycles and changes in light intensity. In cyanobacteria, CO_2_ uptake and HCO_3_^−^ transport are activated within seconds in the light, with CO_2_ uptake preceding HCO_3_^−^ uptake (Badger and Andrews, 1982; Price et al., 2008, 2011b), and both SbtA and BicA are inactivated within seconds in the dark (Price et al., 2013; Förster et al., 2021). While a link between light-activation/dark-inactivation of C_i_ uptake and the state of photosynthetic electron transport and/or to a redox signal has been suggested by Kaplan et al. (1987), the identity of the light signal, signal transduction pathways and sensory/response mechanisms of the C_i_ uptake proteins are still elusive. Furthermore, protein phosphorylation may play a role in post-translational modulation of HCO_3_^−^ transporter activity (Sültemeyer et al., 1998), and it is uncertain whether the native cyanobacterial regulatory kinases/phosphatases could function correctly in plastids when co-expressed with their transporter targets. This level of regulation dependency needs to be addressed to ensure replication of cyanobacterial-like control of C_i_ uptake mechanisms in a C_3_ system.

### Light/Dark Control of C_i_ Uptake

There is some evidence for redox-regulation of CO_2_ uptake by the NDH-1 complexes in cyanobacteria. NDH1_3/4_ function is directly linked to the trans-thylakoid proton motive force and cyclic electron transfer at photosystem I through interaction with ferredoxin and plastoquinone intermediates of the photosynthetic electron transport chain (ETC; Schuller et al., 2020). Light-driven changes in photosynthetic electron transport cause instantaneous changes of the redox state of the ETC which modulates CO_2_ fixation *via* changes in NADPH production, ATP synthesis, and the redox-sensitive activation state of the Calvin-Benson-Bassham (CBB) cycle enzymes. In cyanobacteria, oxidizing conditions activate the small, inhibitory CP12 protein and ferredoxin-thioredoxin redox signaling cascades which inhibit the CBB cycle enzymes (*via* thiol-oxidation of cysteines; McFarlane et al., 2019), thus coordinating CO_2_ uptake and carboxylation. Given that the ETC and the ferredoxin-thioredoxin-CP12 regulatory system are highly conserved and present in all plant chloroplasts, regulatory features may already be present in chloroplasts if large, multi-gene NDH-1 complexes could be heterologously expressed. However, it is unlikely that this modus of redox-regulation applies to plasma membrane-located HCO_3_^−^ transporters, which are spatially separated from the ETC and have not been detected among proteins targeted by thioredoxin (Lindahl and Florencio, 2003).

Currently without experimental evidence, other putative redox-sensitive regulatory mechanisms for cyanobacterial C_i_ uptake, such as eliciting signaling molecules such as Ca^2+^ (Torrecilla et al., 2004; Domínguez et al., 2015), light-stimulated changes in membrane potential (Murvanidze and Glagolev, 1982), and Ca^2+^ sensory phosphorylation relays triggered by light-dark transitions (Mata-Cabana et al., 2012) are speculative. However, regardless of the regulatory mechanism, the main concern remains whether an analogous regulatory system exists in the chloroplast and whether it can interact appropriately with the introduced foreign proteins, or, whether such systems need to be transplanted into chloroplasts alongside C_i_ uptake systems. Importantly, Ca^2+^ plays a major regulatory role for photosynthesis and related metabolism in chloroplasts and light-dark transitions elicit specific Ca^2+^ responses (Pottosin and Shabala, 2016). Therefore, chloroplasts harbor an extensive Ca^2+^ signaling infrastructure and are part of the whole plant signaling network which includes crosstalk between chloroplastic and cytoplasmic Ca^2+^ signaling responses to environmental stimuli (Navazio et al., 2020). How the incorporation of additional systems, which could have Ca^2+^ dependencies, might impact on overall inter- and intra-cellular signaling is yet to be seen.

So far, evidence for control of HCO_3_^−^ uptake involving interaction of the transporter with regulatory proteins and/or additional co-factors has only emerged for SbtA. Heterologous co-expression of SbtA and its cognate P_II_-like SbtB proteins in *E. coli* abolished SbtA-mediated HCO_3_^−^ uptake constitutively and formed SbtA:SbtB containing protein complexes (Du et al., 2014). This suggests activity of SbtA can be modulated through binding its respective SbtB (Fang et al., 2021). Effects on SbtA activity have not been observed in low C_i_-acclimated, SbtB-deficient cyanobacterial mutants (Förster et al., 2021), although initial C_i_ acclimation and growth appeared to be compromised in *Synechocystis* sp. PCC6803 (Selim et al., 2018). However, so far, *in vitro* evidence suggest that certain SbtA and SbtB pairs interact in response to adenylate ratios and adenylate energy charge sensed through SbtB (Kaczmarski et al., 2019; Förster et al., 2021), and even though the *in vivo* role of the SbtA-SbtB interaction is not clear yet, co-expression of SbtA and SbtB may be necessary for appropriate functional control in chloroplasts.

### Implications for pH Balance, Ion Homeostasis, and Energetic Requirements

While single gene HCO_3_^−^ transporters such as the SbtA HCO_3_^−^/Na^+^ symporters are prime candidates for chloroplast expression (Du et al., 2014), accumulation of HCO_3_^−^ and Na^+^ in the stroma in the dark could theoretically lead to pH imbalances and high concentrations of Na^+^ impairing chloroplast biochemistry (Price et al., 2008; Mueller et al., 2014; Myo et al., 2020). Cellular pH is tightly regulated to ensure near optimal conditions for biochemical reactions to occur. The cytoplasmic pH in *Arabidopsis* is maintained at about 7.3 (Shen et al., 2013), whereas the chloroplast stroma has been reported to vary between pH 7.2 in the dark to about pH 8 in the light (Höhner et al., 2016). All membrane systems in plant cells possess numerous transport systems (comprised of cation/H^+^ and anion/H^+^ exchangers) that maintain pH homeostasis in different subcellular compartments, and transmembrane H^+^ gradients as a proton motive energy source. In the light, the capacity for pH-regulation and buffering in chloroplasts is likely to accommodate the alkalinization caused by continued HCO_3_^−^ import into the chloroplast. Bicarbonate accumulation in the chloroplast *via* a single transporter type is unlikely to exceed the pool sizes of up to 50mM measured in CCM-induced and actively photosynthesizing cyanobacteria (Kaplan et al., 1980; Woodger et al., 2005). Moreover, the pH disturbance associated with short-term (~ 5min) exposure of leaves to high CO_2_, which elevated stromal HCO_3_^−^ up to 90mM in the dark and 120mM in the light, was counteracted rapidly within seconds (Hauser et al., 1995). However, it is uncertain whether pH buffering is as effective if continued HCO_3_^−^ uptake in the dark were to accumulate substantial HCO_3_^−^ pools without consumption by Rubisco. Consideration must therefore be given to this uncertainty in CCM engineering strategies.

The second potentially confounding issue with expression of the SbtA and BicA transporters on the chloroplast envelope is the influx of Na^+^. Assuming a stoichiometry of 1:1 for Na^+^ and HCO_3_^−^ co-transport, these transporters could increase chloroplast [Na^+^] by at least as much as the [HCO_3_^−^] mentioned above. In contrast to halophytes which tolerate higher chloroplastic Na^+^ concentrations, photosynthesis in glycophytes (including many C_3_ crop plants) becomes impaired by subtle elevation of stromal Na^+^ from 0.21 to 0.38mM in *Arabidopsis* (Mueller et al., 2014). The NHD1 Na^+^/H^+^ antiporter on the chloroplast envelope is active in Na^+^ extrusion (Figure 2), maintaining a positive Na^+^ gradient for other Na^+^-dependent carriers on the chloroplast envelope, regulating stromal pH, and contributing to salt tolerance (Höhner et al., 2016; Tsujii et al., 2020). This suggests that, in particular, light/dark regulated Na^+^ extrusion and Na^+^/HCO_3_^−^ symport need to be synchronized. Thus, boosting Na^+^ export systems on the chloroplast envelope may be required to restore ion/pH balance, which could involve overexpression of the endogenous NHD1 or expression of foreign Na^+^/H^+^ antiporters such as cyanobacterial NhaS proteins (Price et al., 2013).

Unfortunately, regulation of Na^+^ fluxes between different compartments of plant cells and the characteristics of Na^+^ carriers are not well understood, therefore making it difficult to predict how active HCO_3_^−^ uptake might influence Na^+^ fluxes. In addition to the potential over-accumulation of stromal [Na^+^], it is not clear whether the cytoplasmic [Na^+^] and the magnitude of the Na^+^ gradient across the chloroplast envelope will be sufficient for optimal energization of SbtA or BicA and in varying environments. Estimates of cytoplasmic [Na^+^] range between 3 and 30mM (Karley et al., 2000; Tester and Davenport, 2003), which exceeds the *K*_0.5_ (Na^+^ concentration supporting half-maximum HCO_3_^−^ uptake rates) of 1–2mM Na^+^ for SbtA and BicA (Price et al., 2004; Du et al., 2014). Stromal Na^+^ concentrations have been reported between 0.2 and 7mM (Schröppel-Meier and Kaiser, 1988; Mueller et al., 2014). Therefore, dependent on the plant species and/or environmental conditions, cytoplasmic Na^+^ is in the lower concentration range, and the differential between cytoplasm and stroma, could impose limits on HCO_3_^−^ uptake rates depending on substrate availability. However, plants under field conditions experience relatively higher salinity in most agricultural soils than in controlled growth environments, which means we can expect their cells operate at slightly elevated cytoplasmic Na^+^ levels (Tester and Davenport, 2003), which renders Na^+^ limitation fairly unlikely.

Based on homology to ABC transporters, the cyanobacterial BCT1 and the *Chlamydomonas* HLA3 (Figures 1A,C) are thought to be energized by ATP hydrolysis, but the ATP required per HCO_3_^−^ transported has not been determined. Modeled ATP requirements for SbtA and BicA activity, which consume ATP indirectly as costs for proton transport to maintain the Na^+^ gradient, project 0.5 and 0.25 ATP, respectively, per HCO_3_^−^ transported (Price et al., 2011a). Particularly at low external C_i_, suppression of photorespiration by active HCO_3_^−^ uptake is more ATP cost-effective than typical C_3_ photosynthesis, and ATP demand for transporter function should be readily covered by photophosphorylation in the chloroplast. The modeling did not consider additional ATP requirements for synthesis and maintenance of C_i_ uptake complexes though, since protein accumulation and turnover rates are unknown in both native organisms and chloroplasts, which is a modest pressure onto ATP production compared to the overall daily expenditure in living cells. Recent modeling of proposed pyrenoid-based systems also highlights ATP costs to chloroplastic CCMs; however, these can be limited depending on the engineering strategy (Fei et al., 2021).

## How can we get C_i_ Uptake Systems Into the Chloroplast?

The expression of transgenes from the nuclear genome of terrestrial plants is the favored means to introduce a CCM into crop plants due to current difficulties associated with successful insertion of exogenous genes into the chloroplast genomes of some major crops (Hanson et al., 2013). Nonetheless, many proof-of-concept approaches utilize plastome expression to assess CCM components (Lin et al., 2014b; Pengelly et al., 2014; Long et al., 2018). We focus here on strategies relating to the import of nuclear-encoded proteins into chloroplastic membranes and stroma where broader application to the majority of globally important crops is feasible. This approach introduces many complicating challenges when considering the transfer of systems from a cyanobacterium where proteins are targeted to the membrane from the inside, whereas in chloroplast proteins would come from the outside.

Successful transport of HCO_3_^−^ into C_3_ plant chloroplasts requires that a transporter will be pumping solute across the chloroplast IEM, into the chloroplast stroma. This sounds simple in principle but implies several assumptions about the transporter are true. Firstly, that it is successfully expressed and targeted to the chloroplast. Secondly, correct direction of the imported protein to the chloroplast IEM occurs. Thirdly, the protein must fold and orient itself in the appropriate manner such that its intended direction of transport is inward to the stroma. Finally, any processes which ensure activation and energization of the transporter must be met (discussed above). Correct targeting of HCO_3_^−^ transporters to the chloroplast IEM has been the subject of several reports in recent years (Atkinson et al., 2016; Rolland et al., 2016, 2017; Uehara et al., 2016, 2020; Nölke et al., 2019), however, correct localization, orientation, and activation of these proteins to ensure favorable function remain an engineering challenge.

### Foreign Protein Expression and Targeting

The initial step of expressing foreign genes in transgenic plants is a common point of failure due to a myriad of factors relating to gene positional effects (Pérez-González and Caro, 2019) and silencing (Jackson et al., 2014), codon usage (Nakamura and Sugiura, 2009), promotor and terminator combinations (Beyene et al., 2011; de Felippes et al., 2020), and potential degradation of the precursor protein (Lee et al., 2009; Shen et al., 2017; Hristou et al., 2020). This usually requires the analysis of relatively large numbers of plant transformation events and somewhat laborious testing of gene expression cassettes (often in transient expression systems) to ensure appropriate levels of protein expression can be achieved. We do not provide further discussion on this point but highlight that fine-tuning this aspect of CCM engineering in C_3_ plants is not trivial and can heavily impact on the trajectories of engineering approaches.

**Figure 3:**
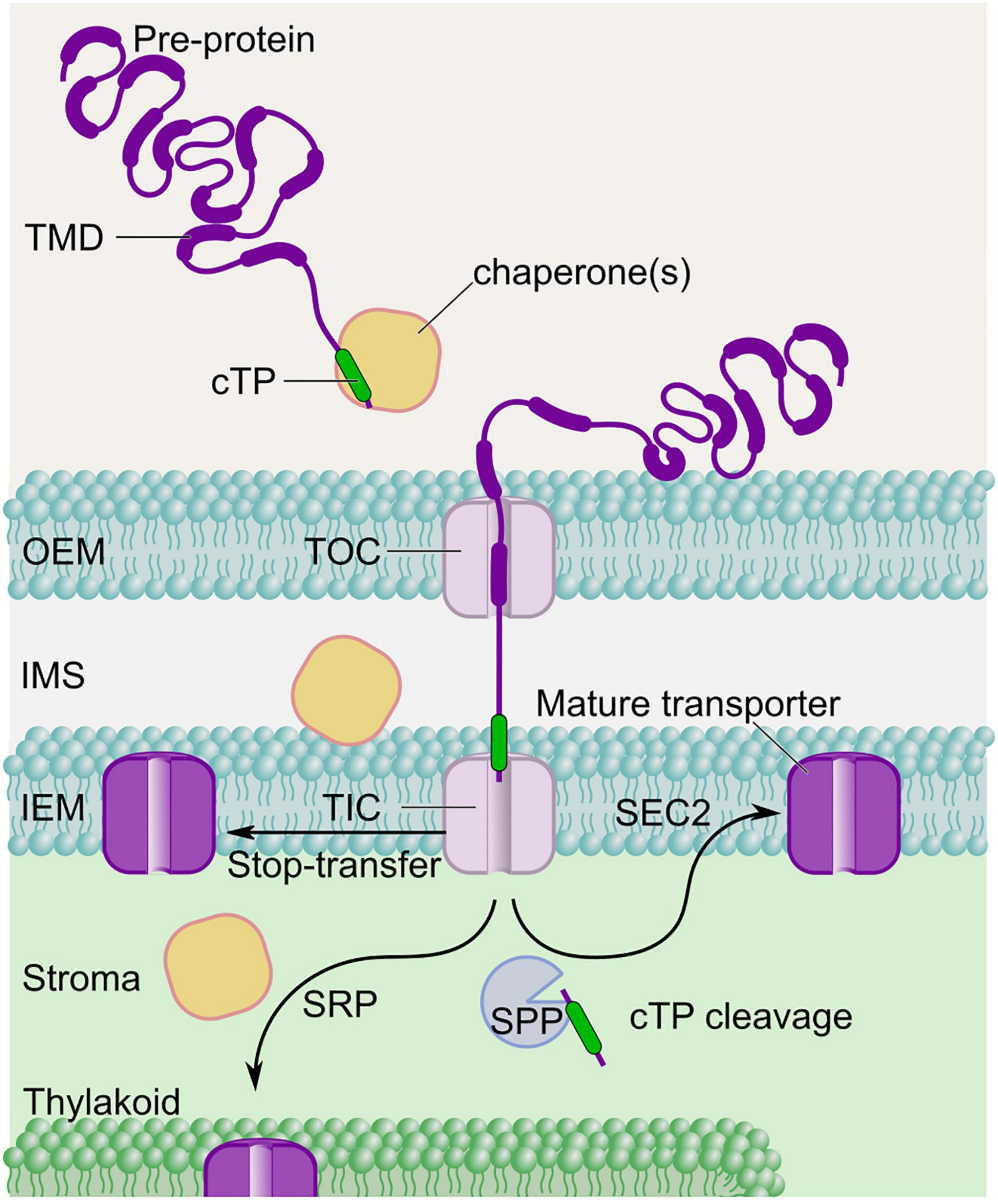
Possible pathways for membrane HCO_3_^−^ transporter insertion in C_3_ chloroplasts. For successful targeting to the correct membrane in C_3_ chloroplasts, candidate inorganic carbon uptake system proteins need to utilize an appropriate chloroplast targeting peptide (cTP) at the N-terminus of the nuclear-encoded pre-protein. Chaperones co-ordinate transit of membrane pre-proteins to the TIC-TOC translocons enabling initial import into the chloroplast. Here, proteins can undergo a number of localization processes, dependent on sequence motifs and interactions. Proteins destined for the IEM are handled by the stop-transfer or post-import (SEC2) pathways, whereas the signal recognition particle (SRP) pathway is utilized by proteins destined for the thylakoid (Lee et al., 2017). Ultimate localization can be partly driven by transmembrane domain (TMD) sequences (Rolland et al., 2017; Singhal and Fernandez, 2017). The stromal processing peptidase (SPP) must recognize and cleave the cTP in such a way that no functionally disruptive scar is left behind on mature transporters when they reach their proper destination. Very little is known about targeting foreign proteins to the IMS.

Once expressed, nuclear encoded proteins targeted to the chloroplasts are translocated as pre-proteins within the cytosol where chaperones, such as Hsp70, Hsp90, and the 14–3-3 protein complex are involved throughout the translocation process (Figure 3; May and Soll, 2000; Schwenkert et al., 2011). Proteins translated in the cytosol and destined for the chloroplast either remain unfolded with the help of chaperones (Jarvis, 2008), or can be imported to the chloroplast in a fully-formed state (Ganesan et al., 2018), prior to translocation across the chloroplast envelope. These chaperones are crucial to prevent the premature folding of large proteins and aggregation and/or degradation of pre-proteins (Wojcik and Kriechbaumer, 2021).

Upon reaching the chloroplast, pre-proteins enter through the TIC-TOC pathway and are then directed within the chloroplast to their final destination (e.g., IEM, OEM, stroma, thylakoid membrane, or lumen; Figure 3; Oh and Hwang, 2015; Lee et al., 2017; Xu et al., 2020). Noticeably, post-import insertion into the IEM could involve additional processing by the Cpn60/Cpn10 chaperonin complex within the stroma, prior to insertion into the IEM through a membrane bound translocase (SEC2; Li and Schnell, 2006; Li et al., 2017). These various processes are facilitated by the pre-protein chloroplast transit peptide (cTP) which possesses binding sites for chaperones and is crucial for targeting nuclear-encoded proteins into the chloroplast (Ivey et al., 2000; Rial et al., 2000; Lee and Hwang, 2018). Therefore, the types of chaperones that would mediate foreign pre-protein chloroplast import would depend on the cTP used. There is currently no understanding of the cyanobacterial chaperone requirements for CCM-related HCO_3_^−^ transporters in their native systems, thus we must rely on host system chaperones for correct folding (if required) in heterologous plant expression. However, Atkinson et al. (2016) and Nölke et al. (2019) have shown successful targeting of microalgal chloroplast membrane transporters, suggesting there is a propensity for direct transfer of proteins from homologous systems with chloroplasts. Notably, the common ancestral origin of cyanobacteria and C_3_ plant chloroplasts is partly identified in shared phylogeny of many of their outer membrane proteins (Day and Theg, 2018), and this might suggest potential for successful transfer of cyanobacterial membrane components to the chloroplast IEM. However, the transfer of genes from the plastome to the nucleus during C_3_ plant evolution means both the inversion of directional insertion of membrane proteins (Day and Theg, 2018), and the emergence of cTPs to enable protein trafficking through the TIC-TOC complex and to the correct membrane (Figure 3; Knopp et al., 2020; Ramundo et al., 2020).

Correct targeting to the chloroplast membranes is further complicated by the presence of additional organelles in plant cells, and dual targeting between chloroplasts and mitochondria is commonly observed (Peeters and Small, 2001; Sharma et al., 2018). This complexity of organelle targeting (Bruce, 2000; Wojcik and Kriechbaumer, 2021) requires specific choice of cTP in proposed photosynthetic engineering strategies, and we suggest that the direction of foreign proteins to the appropriate cellular compartment is unlikely to be a one-size-fits-all solution (Rolland et al., 2017). There are also likely to be protein cargo-specific requirements which determine the choice of cTP for each heterologous membrane protein directed to the chloroplast IEM, thus identifying the need to test and tailor genetic constructs on an individual basis. This strategy is also required to optimize promotor/terminator requirements and is highly relevant in systems where protein stoichiometry (such as for multi-protein complex transporters such as BCT1) may be essential for function.

Successful incorporation of multi-component membrane transporter complexes such as BCT1 (Figures 1, 2) will require subunits which lie not only in the IEM, but also in the IMS and the stroma of the chloroplast. Targeting to the IMS has not been well investigated, with few examples in the literature investigating the subject (Kouranov et al., 1999; Vojta et al., 2007). At least two pathways to this location are thought to exist, one where proteins mature in the IMS (e.g., the TIC complex subunit Tic22; Kouranov et al., 1999), and one where proteins transit through the stroma and are then re-inserted into the IMS (e.g., MGD1; Vojta et al., 2007). Which may be the most appropriate pathway and whether foreign proteins can utilize either approach is yet to be described. In contrast, targeting to the stroma has been thoroughly studied and might therefore be the easiest to achieve (reviewed in Li and Chiu, 2010). One aspect worth mentioning is the stromal processing peptidase (SPP) which is known to cleave cTPs from several nuclear-encoded proteins imported into the stroma (Figure 3; Richter and Lamppa, 1998, 2002). The complete removal of cTPs is highly desirable in chloroplast engineering, as N-terminal additions to foreign proteins can impede their function. However, successful cTP cleavage may be prevented by cargo protein secondary and tertiary structure. With difficult cargoes, cTPs may need to be extended beyond the cleavage site with a flexible linker which will ultimately leave a scar that might also impede protein function. Notably, however, some novel cTPs have been designed to reduce the proteolytic scars while enhancing targeting of difficult protein cargoes. These engineered cTPs, such as RC2 and PC1 (Shen et al., 2017; Yao et al., 2020) include about 20 residues from its native mature cargo (a spacer to allow translocating factors better access to the cTP) which are followed by a second SPP cleavage site (to allow removal of the additional 20 residues used as spacer). Another approach that has been specifically used for the HCO_3_^−^ transporters SbtA and BicA included a TEV protease cleavage site after the cTP to enable removal by a heterologously expressed TEV protease (Uehara et al., 2016, 2020).

As mentioned above, the NDH complex may depend on plastoquinone for energization, and if we were to use such a complex for CO_2_ recapture, the chloroplast thylakoid membrane would be the destination of choice (Figure 2; Long et al., 2016; Price et al., 2019; Hennacy and Jonikas, 2020). While the chloroplast twin arginine translocation, and secretory pathways direct mostly soluble proteins to the thylakoid lumen, it is the chloroplast signal recognition pathway (SRP) that targets membrane proteins to the thylakoid membrane (Figure 3; Smeekens et al., 1985; Schnell, 1998; Aldridge et al., 2009; Ouyang et al., 2020; Xu et al., 2020). Note that dual targeting between thylakoid and IEM was encountered when foreign transporters were targeted to the IEM (Pengelly et al., 2014; Rolland et al., 2017). A study on two closely related *Arabidopsis* proteins, SCY1 (thylakoids) and SCY2 (IEM), shed light on the sorting mechanism between IEM and thylakoids. In brief, the N-terminal region of SCY2 alone was not sufficient for exclusive targeting to the IEM. Instead, two internal transmembrane domains (TMDs) were required to achieve unambiguous localization to the IEM with no leakage toward the thylakoid membrane (Singhal and Fernandez, 2017). This study demonstrated that targeting is cargo-dependent. Hence, a more complex engineering of cargo TMDs might be required to successfully target foreign HCO_3_^−^ transporters within the chloroplast (Rolland et al., 2017).

### Control of Membrane Protein Orientation

Due to the inverted targeting strategy proposed for cyanobacterial transporters, there is potential for nuclear-encoded membrane proteins to be incorrectly oriented in the chloroplast IEM, even if targeting is successful. Most of the work done to understand membrane protein orientation (i.e., TMD topology) has been carried out in bacteria, establishing the positive-inside rule (Lys and Arg rich loops orient in the cytoplasm; von Heijne, 1986) and the charge-balanced rule (Dowhan et al., 2019). However, little is known about topology determinants in C_3_ plant chloroplast membranes. Membrane lipid composition is known to influence the orientation of membrane proteins in the OEM (Schleiff et al., 2001). However, since the lipid composition of the C_3_ chloroplast OEM differs from the IEM (Block et al., 2007), it is difficult to draw parallels between their orientation determinants. Interestingly, specific TMDs also appear to affect membrane protein orientation (Viana et al., 2010; Okawa et al., 2014). While changing lipid composition to control orientation is unrealistic in plants (but was achieved in bacteria; Dowhan et al., 2019), rational design of TMDs, and interconnecting loops (Rapp et al., 2007) from HCO_3_^−^ uptake systems might be an option. As shown for the secretory pathway in plant endoplasmic reticulum, membrane protein signal peptides may also play a role in the orientation of some proteins (Wojcik and Kriechbaumer, 2021). Hence, it is reasonable to assume that correct targeting and orientation of membrane proteins in the chloroplast IEM are dependent on both the cargo protein and its targeting sequence (Rolland et al., 2016; Uehara et al., 2016, 2020). As a result, broad screening of targeting peptides for candidate cyanobacterial membrane protein cargos is likely required, both on a case-by-case basis and possibly between heterologous hosts. Membrane protein orientation must therefore be considered when addressing CCM component expression in plant systems and will affect predicted outcomes of functional HCO_3_^−^ uptake assessment in transformed plants.

## Perspectives and Conclusion

Application of synthetic biology approaches to elevate HCO_3_^−^ concentrations in C_3_ plant chloroplasts, as a means to enhance Rubisco carboxylation, is an ongoing engineering endeavor among plant biologists. It is, however, a complex task which needs to be considered within a broad framework of molecular and physiological complexity. Efforts to heterologously express candidate HCO_3_^−^ transporters and CO_2_-to-HCO_3_^−^ converting complexes in C_3_ plants must therefore be contemplated within this context. Therefore, it is critically important that researchers addressing this challenge gather evidence of correct targeting, orientation and processing of protein transporters in plant systems. Functionality should be addressed where possible, and techniques which provide evidence of successful HCO_3_^−^ import (e.g., Tolleter et al., 2017) and elevated leaf-level carboxylation should accompany reports of plant growth and productivity to ensure that predicted physiological outcomes correlate with enhanced growth. In addition to this, greater detail is required on the functional characterization of existing HCO_3_^−^ uptake systems in their native systems (Table 1), while an understanding of the broader natural variation in HCO_3_^−^ uptake systems (e.g., Scott et al., 2018; Desmarais et al., 2019) should be accumulated to provide greater options for engineering purposes.

## Author Contributions

BF generated the table. BML generated the figures. All authors contributed to the article and approved the submitted version.

## Conflict of Interest

The authors declare that the research was conducted in the absence of any commercial or financial relationships that could be construed as a potential conflict of interest.

## Publisher’s Note

All claims expressed in this article are solely those of the authors and do not necessarily represent those of their affiliated organizations, or those of the publisher, the editors and the reviewers. Any product that may be evaluated in this article, or claim that may be made by its manufacturer, is not guaranteed or endorsed by the publisher.
